# Efficient Estimation of Permeate Flux of Asymmetric Ceramic Membranes for Vacuum Membrane Distillation

**DOI:** 10.3390/molecules27031057

**Published:** 2022-02-04

**Authors:** Kaiyun Fu, Yunzhao Guo, Wenbo Qi, Xianfu Chen, Minghui Qiu, Yiqun Fan

**Affiliations:** State Key Laboratory of Materials-Oriented Chemical Engineering, College of Chemical Engineering, Nanjing Tech University, Nanjing 211816, China; fukaiyun@njtech.edu.cn (K.F.); yzguo@njtech.edu.cn (Y.G.); 202061204258@njtech.edu.cn (W.Q.); qiumh_1201@njtech.edu.cn (M.Q.); yiqunfan@njtech.edu.cn (Y.F.)

**Keywords:** ceramic membrane, vacuum membrane distillation, permeate flux, modeling

## Abstract

Ceramic membranes have the advantages of high mechanical strength and thermal stability and are promising candidates for membrane distillation. Ceramic membranes are generally designed to have a multilayer structure with different pore sizes to create a high liquid entry pressure and obtain a high permeability. However, these structural characteristics pose significant difficulties in predicting permeate flux in a ceramic membrane contactor for vacuum membrane distillation (VMD). Here, a modeling approach was developed to simulate the VMD process and verified by comparing the simulated results with the experimental data. Furthermore, correlations are proposed to simplify the calculations of permeate flux for VMD using asymmetric ceramic membranes by assuming those multilayers to be an effectively quasi-symmetric layer and by introducing a correction factor. The simulation results indicated that this simplified correlation was effective and enabled a quick estimation of the effect of membrane parameters on permeate flux.

## 1. Introduction

Membrane contactors have received considerable attention in various applications such as membrane distillation (MD) for desalination [1,2], membrane absorption (MA) [3], and membrane stripping (MS) [4] for CO_2_ capture, due to their high mass transfer area and flexible installation and operation. Four configurations of MD processes have been extensively investigated for separation, including direct contact membrane distillation (DCMD), air gap membrane distillation (AGMD), sweep gas membrane distillation (SGMD), and vacuum membrane distillation (VMD). VMD could provide a higher mass flux than other MD configurations and has received considerable attention [5].

The membranes for MD application are expected to be porous and hydrophobic and have high thermal stability and chemical resistance to feed solutions [1]. Compared with polymeric membranes, ceramic membranes, such as alumina (Al_2_O_3_) and zirconia (ZrO_2_) membranes, benefit from higher mechanical strength and thermal stability [6,7] and are suitable for application under harsh operating conditions. They have shown promising potential for membrane absorption [8,9,10], membrane reaction [11], membrane distillation [12], and other applications. Typical ceramic membranes are usually designed to have an asymmetric structure, so to combine high flux and high selectivity. They can be divided by functionality into a support layer, a top layer, and one or more transition layers located between the two aforementioned layers [13]. The support layer is prepared as a wall with millimeter thickness and macro pores, providing high mechanical strength and mass flux. The top layer is a thin membrane with micrometer thickness and small pores in the range of tens to hundreds of nanometers, creating the required counterpressure or functioning as a selective barrier. The transition layers have pores with medium size, between those of the pores in the support and top layers. The membrane materials used for VMD are mostly hydrophobic. Since hydrophobic pores prevent the entrance of the feed solution, only vapor is able to pass through the pores. Pores filled with gas or vapor usually have higher mass transfer performance compared with those filled with liquid, since the VMD process is driven by transmembrane pressure and temperature differences. Although ceramic membranes are inherently hydrophilic, they can be functionalized to achieve a hydrophobic character for application in MD [2,14].

Gas transport in a porous membrane material is a microscopic or molecular process that is more complicated than the macroscopic or bulk transport of a gas fluid [15]. In the VMD process for desalination, water, as a volatile component evaporates at the feed side of the membrane and then is transported through the membrane pores into the permeate side. Several mechanisms are possible for the transport of gases through porous membrane(s) and supports, separate or combined. Which mechanism dominates the transport depends on certain conditions of pressure and temperature and on the specific pore size range. In general, the transport of gases through a membrane is mostly affected by pore size, followed by pressure and temperature. In the VMD process, vapor is transported through an asymmetric ceramic membrane at a variable transport coefficient since the pore sizes of different layers vary greatly. Therefore, it is more complex and difficult to estimate the permeate flux of a multilayer membrane than that of a symmetric membrane. In the case that the transport coefficient is only affected by the membrane structure and not by pressure and temperature, the overall gas flux of an asymmetric membrane could be calculated with a mass transfer resistance-in-series approach. Because of the practical difficulty in measuring pressure and temperature within the membrane pores, the calculation of transport flux requires an initial guess of the interfacial pressures and temperatures and multiple iterations until the fluxes of all layers are equal [16,17]. Therefore, developing an effective approach that avoids the need to perform an iterative computation of the gas transport flux for asymmetric membrane is essential for the design and optimization of special ceramic membrane elements. However, improved approached for predicting the permeate flux of asymmetric membranes for VMD can rarely be found in the open literature.

In this work, a comprehensive 2D modeling approach was applied to simulate gas transport through a tubular asymmetric membrane. The simulation results were compared with the VMD experimental data obtained in this work using five specifications of tubular α-Al_2_O_3_ ceramic membranes which were modified to be hydrophobic. Furthermore, a novel approach that considers the equivalent effects of the structure properties (i.e., membrane thickness and pore size) of different layers on the overall transport flux is proposed for a practical and efficient prediction of permeation in the VMD process.

## 2. Results and Discussion

### 2.1. Validation of the Modeling Approach

**Figure 1:**
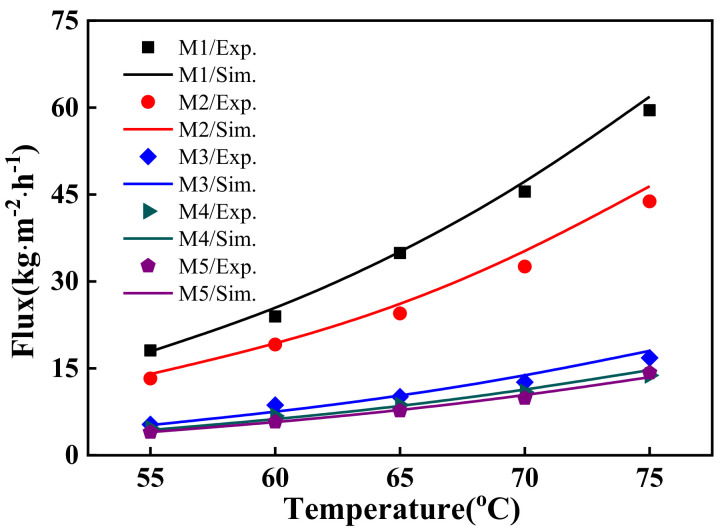
Comparison between the experimental and the predicted water flux.

To validate the proposed model for the VMD process, simulations were performed for the five membrane tubes and then validated with experimental data. The comparison between the measured and the predicted permeate flux in different temperature and pressure conditions are presented in Figure 1. Clearly, the proposed model offered high prediction accuracy, with an average absolute deviation (AAD), which can be defined with Equation (1), of less than 3.4%.

(1) $$\mathrm{AAD}=\frac{1}{N}\overset{N}{\underset{i=1}{\sum }}\left|\frac{Sim_{i}-Exp_{i}}{Exp_{i}}\right|$$

After model validation, more simulations can be performed for wider ranges of operational conditions and membrane characteristics, which are beneficial for the analysis of gas permeate characteristics in a multilayer ceramic membrane.

### 2.2. Effects of Several Key Parameters on Permeate Flux

**Figure 2:**
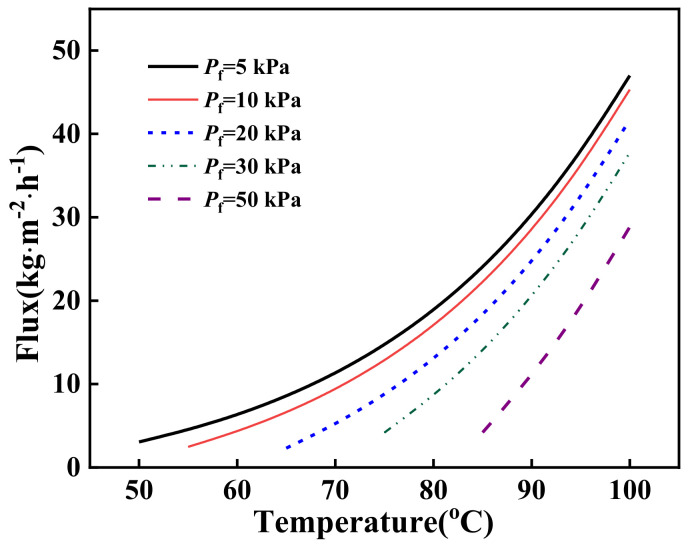
Effects of pressure and temperature on water permeate flux.

To investigate the influences of temperature and pressure on vapor transport through asymmetric ceramic membranes, simulations were performed using M5. As shown in Figure 2, the permeate flux of water increased as the vacuum degree and liquid temperature increased. These phenomena were ascribed to the increase of transmembrane pressure difference, represented in Equation (9), since an increase of the vacuum degree will lower the permeate side pressure, and an increase of the feed temperature will increase the saturated vapor pressure of water.

**Figure 3:**
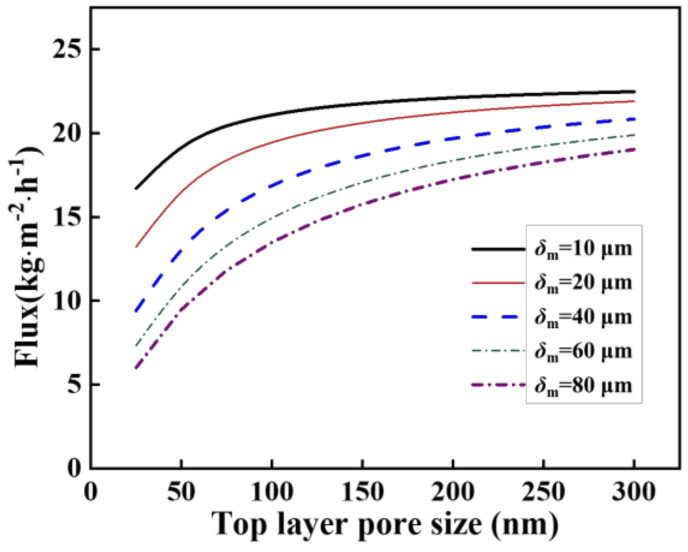
Effect of pore size and thickness of the top layer on water flux.

In addition to temperature and pressure, membrane parameters play important roles in gas transport through a membrane. Here, simulations were performed for various asymmetric membranes which had the same support structures (with pore size of 1.0 μm and thickness of 2.0 mm) but different top layer parameters. The effect of the pore size in the top layer on the vapor permeate flux for membranes with various top layer thicknesses can be seen in Figure 3. As illustrated, the permeate flux decreased as the thickness of the top layer increased. Moreover, the permeate flux precipitously increased as the pore sizes increased, when the pore size was smaller than 50 nm. This occurred because an increase in pore size is a great advantage for improving Knudsen diffusion and Poiseuille flow (Equation (9)). Subsequently, the increase of permeate flux became slow as the pore size of the top layer further increased. This phenomenon can be explained by the contribution of the top layer to the overall mass transfer resistance that decreased gradually with the increase of the top layer pore size.

**Figure 4:**
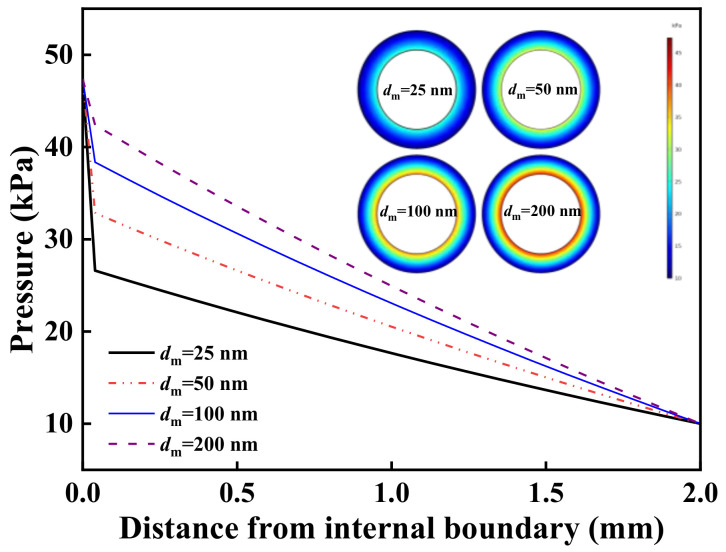
Cross-sectional pressure distribution of various asymmetric membranes (the thickness of the top layer was 40 μm, the pore size of the support layer was 1.0 μm).

The contours of static pressure distribution for different membranes were simulated to analyze the distribution of mass transfer resistance of asymmetric membranes. As shown in Figure 4, when the pore size of the top layer was very small, the portion of pressure drop across the top layer was very high, indicating that the top layer dominated the overall mass transfer process even though it was very thin. With the increase of the top layer pore size, the mass transfer controlling step turned from the top layer to the support layer.

With the above simulation results, it is reasonable and easy to relate the water flux to temperature, pressure, pore sizes, and thickness of asymmetric membranes. However, the results are insufficient for designing a practical membrane contactor and for optimizing related membrane parameters and operational conditions. A successful design of a membrane element with appropriate properties can be considered as a multi-objective optimization problem with multiple constraints, including at least its ability to create a high liquid entry pressure (LEP), permeability, mechanical strength, and current preparation level of the membrane material. For example, the pore size of the skin layer of a used membrane should be smaller than the maximum value of LEP, according to the Laplace–Young relationship [18]. In addition, the used membrane should be thick enough to provide enough mechanical strength. Considering these requirements, increasing the pore size of the support layer is a good approach to improve the overall permeability of a membrane element. If the pore sizes of the top and support layers differ greatly, an increase in the thickness of the top layer or the introduction of one or more transition layers with medium average pore size between the two layers is required to enable an effective coverage of the support layer by the nanoparticles on the skin layer. However, these changes will increase mass transfer resistance.

As can be seen in Equation (9), the functional relationship between the positive effect of pore size and the negative effect of thickness on the permeate flux is not simple. Thus, the following question arises: how to reach an optimal trade-off between the increase of the pore sizes of the support layer and that of the thickness of the top and transition layers? Moreover, since the transmembrane flux of each layer is greatly related to its temperatures and pressures on both sides, the calculation of overall permeate flux for an asymmetric membrane requires an iterative procedure, assuming intermediate parameters and calculating the corresponding flux until the flux of each layer becomes equal. Consequently, the calculation of permeate flux is complicated and impractical.

### 2.3. Simplified Diffusion Correlation

**Figure 5:**
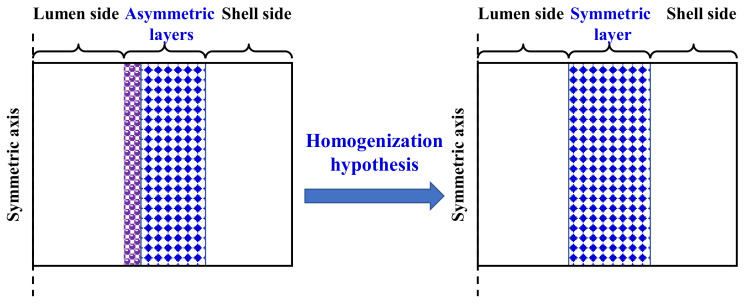
Schematic diagram of a quasi-homogeneous membrane.

The calculation of permeate flux through an asymmetric membrane in VMD is greatly simplified if the transfer equations and boundary conditions could be reasonably reduced. To achieve this, a simplified and practical diffusion correlation for an asymmetric membrane is indispensable. In the development of such a diffusion model, some assumptions were made as described below. An asymmetric membrane, consisting of two or more layers, was considered to be a quasi-homogeneous membrane which was assumed to have the same overall thickness, porosity, and tortuosity as an asymmetric membrane and the same pore size as that of the support layer of the asymmetric membrane, as illustrated in Figure 5.

These assumptions will undoubtedly lead to a higher permeate flux of the quasi-symmetric membrane with respect to that of the asymmetric membrane. A feasible approach to eliminate this positive deviation consists in introducing into Equation (9) a correction factor (*η*) of less than 1. Accordingly, a model for the permeate flux of the quasi-homogeneous membrane can be expressed as follows:

(2) $$J=\eta \left(\frac{d_{p}\varepsilon }{3RT\tau \delta }{\left(\frac{8RT}{\pi M}\right)}^{0.5}+\frac{d_{p}^{2}\varepsilon }{32\mu RT\tau \delta }P_{\mathrm{ave}}\right)\Delta P$$

Here, two problems appear: (i), how to determine the correction factor *η* and (ii), how to associate *η* with various variables related to operating conditions and membrane parameters while ensuring that it retains reasonable physical meaning. The process of obtaining an *η* value can be described as follows. A set of two simulations was performed for an asymmetric membrane and its corresponding quasi-homogeneous membrane under the same operating conditions. Then, an optimizing parameter estimation was employed with *η* representing a variable parameter and with the value of water flux obtained from the former simulation as the targeted value of the latter estimation. After that, the desired *η* value could be obtained. Based on the simulation results in Section 2.2, *η* should obviously be a function of various membrane and operating parameters, including pore size, membrane thickness, temperature, pressure, etc.

To determine *η* and to establish its functional expression, simulations were performed for two kinds of asymmetric membranes (one consisting of two layers, and the other with three layers) with various membrane properties at different operating conditions, as presented in Table 1. To begin in a simple manner, the investigation of *η* was first carried out for a two-layer membrane and then extended to a three-layer membrane.

Since *η* should be less than 1, an *η_two_* correlation for a two-layer membrane was proposed in terms of a rational function expressed by dimensionless numbers.

(3) $$\eta_{two}=\frac{1}{1+a{\left(d_{sup}^{\Theta }\right)}^{b}{\left(d_{top}^{\Theta }\right)}^{c}{\left(\delta_{top}^{\Theta }\right)}^{d}}\;d_{sup}^{\Theta }=\frac{d_{sup}}{d_{ref}};\;d_{top}^{\Theta }=\frac{d_{top}}{d_{sup}};\;\delta_{top}^{\Theta }=\frac{\delta_{top}}{\delta_{overall}}$$

where $d_{\sup}^{\Theta }$, $d_{\mathrm{top}}^{\Theta }$, and $\delta_{\mathrm{top}}^{\Theta }$ are dimensionless membrane parameters; *d_sup_* and *d_top_* are the pore sizes of the support layer and top layer, respectively, *d_ref_* is a reference pore size (1 nm) used for the dimension; *δ_top_* and *δ_overall_* are the thicknesses of the top layer and the of the overall asymmetric membrane; *a* ~ *d* are constants.

The three dimensionless numbers were proposed in consideration of quantifying the equivalent mass transfer resistance between the decrease of top layer’s pore size and the increase of its thickness. Through data fitting, a set of optimum values of *a* ~ *d* in Equation (3) was found to be 0.036, 0.55,−1.08, and 0.95, respectively. Thus, *η_two_* can be written as in Equation (4):

(4) $$\eta_{two}=\frac{1}{1+0.036{\left(d_{sup}^{\Theta }\right)}^{0.55}{\left(d_{top}^{\Theta }\right)}^{-1.08}{\left(\delta_{top}^{\Theta }\right)}^{0.95}}$$

**Figure 6:**
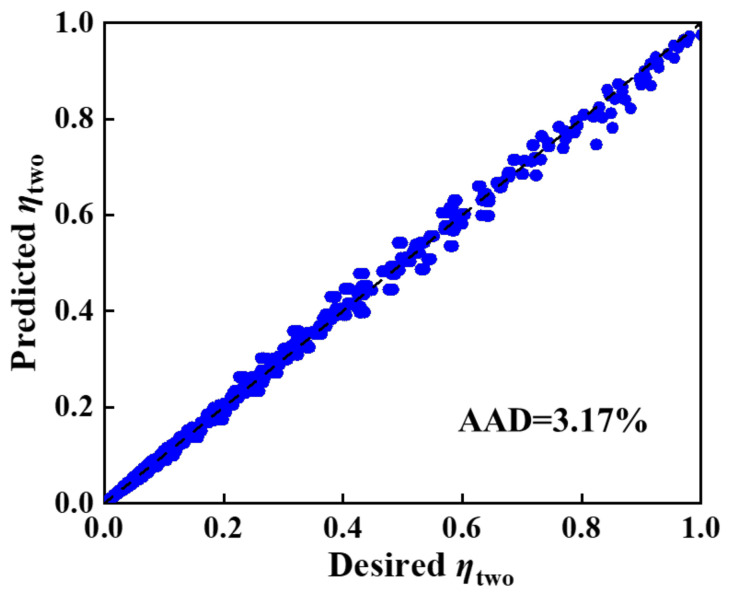
Crossplot between desired and predicted *η* values for two-layer membranes.

The predicted *η_two_* values were plotted versus the desired *η_two_* (obtained from the simulation results), as shown in Figure 6. It was found that the predicted results were in relatively good agreement with the desired ones, with an AAD of 3.17%, which indicated that Equation (4) has high fitting degree and applicability for a two-layer membrane.

Subsequently, *η_two_* was extended to obtain a derivation *η_three_* for a three-layer membrane. With the addition of a transition layer, pore size and thickness can also affect the overall permeability of an asymmetric membrane. The parameter *η_three_* was proposed in relation to of an in-series model, as shown in Equations (5):

(5) $$\eta_{three}=\frac{1}{1+0.074{\left(d_{sup}^{\Theta }\right)}^{0.483}\left[{\left(d_{top}^{\Theta }\right)}^{-0.944}{\left(\delta_{top}^{\Theta }\right)}^{0.933}+{\left(d_{tran}^{\Theta }\right)}^{-1.07}{\left(\delta_{tran}^{\Theta }\right)}^{1}\right]}\;d_{sup}^{\Theta }=\frac{d_{sup}}{d_{ref}};\;d_{top}^{\Theta }=\frac{d_{top}}{d_{sup}};\;d_{tran}^{\Theta }=\frac{d_{tran}}{d_{sup}};\delta_{top}^{\Theta }=\frac{\delta_{top}}{\delta_{overall}};\delta_{tran}^{\Theta }=\frac{\delta_{tran}}{\delta_{overall}}$$

where $d_{\mathrm{tran}}^{\Theta }$, $\delta_{\mathrm{tran}}^{\Theta }$, *d_tran_*, and *δ_tran_* are dimensionless pore size and thickness and the pore sizes and thickness of the transition layer, respectively; *d_ref_* is a reference pore size (1 nm).

**Figure 7:**
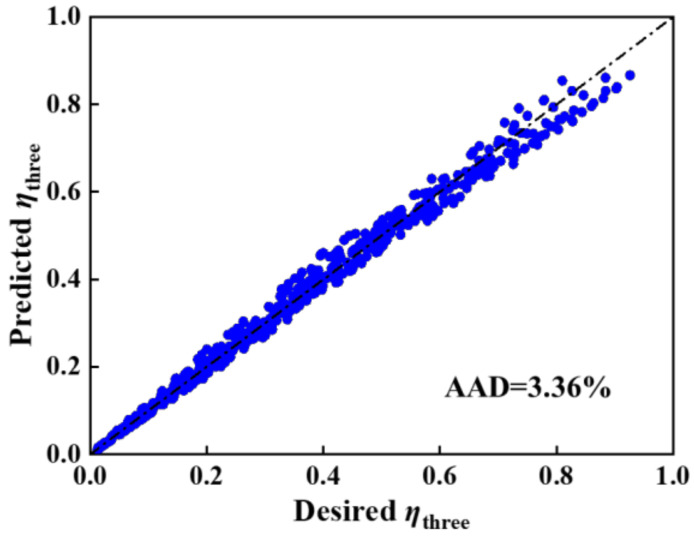
Crossplot between desired and predicted *η* values for three-layer membranes.

To check against the applicability of the predictive correlation of *η_three_*, more than 1000 simulation runs were performed, considering various membrane parameters and operating conditions. Figure 7 shows that the predictive *η_three_* results were in relatively good agreement with the desired *η_three_* values, with an AAD of 3.36%, which indicated that Equation (5) were effective.

It should be noted that pore wetting, which refers to the presence of liquid inside the pores, may occur in MD processes, affecting vapor transport in asymmetric membranes [19]. To make this model more general and robust, more experimental and simulation studies on the pore wetting phenomenon in MD processes and its effect on vapor transport are critical.

## 3. Materials and Methods

### 3.1. Mass and Heat Transfer for VMD

So far, several transport mechanisms have been proposed and demonstrated for the transport of gases or vapors through porous membranes, including ordinary molecular diffusion, Knudsen diffusion, Poiseuille flow, surface diffusion, and/or their combinations [15]. In the case of the VMD process, continuous vacuum is applied on the permeate side to remove vapor trapped in the pores, then the molecular diffusion resistance is generally neglected [20]. In addition, because of the hydrophobic nature of the membranes, the molecule–surface interaction will be low, so surface diffusion also can be ignored [21]. Consequently, the VMD mass flux can be described by Knudsen diffusion, Poiseuille flow, or their combination [16,22,23,24,25]. The mechanism explaining the transport behavior of specie(s), considering a given pore under given operating conditions, mainly depends on the Knudsen number (*Kn*), which can be calculated as follows:

(6) $$K_{n}=\frac{\lambda }{d_{p}},\;\lambda =\frac{k_{B}T}{\pi \sqrt{2}P\sigma^{2}}$$

where *λ* is the mean free path of the gas molecules, m, *d_p_* is the average pore diameter, m; *k*_B_ is the Boltzmann constant (1.3807 × 10^−23^ J/K), *T* is the temperature, K, and *σ* is the molecular collision diameter, m.

When *Kn* < 0.05, molecule–pore wall collisions control the gas transport mechanism, and the gas flux can be calculated as:

(7) $$J=\frac{d_{p}\varepsilon }{3RT\tau \delta }{\left(\frac{8RT}{\pi M}\right)}^{0.5}\Delta P$$

where *J* is the gas flux, mol/(m^2^∙s), *d_p_* and *δ* are the average pore size and membrane thickness, respectively, m, *ε* and *τ* are porosity and tortuosity; *μ* is the viscosity of the gas, kg/(m∙s), *R* is a thermodynamic constant, 8.314 J/(mol∙K), *T* is gas temperature, K, *M* is the molecular weight of the gas, kg/mol, and Δ*P* is the transmembrane pressure, Pa.

When *K*n > 50, molecular–molecular collisions dominate, and mass transfer can be calculated as:

(8) $$J=\frac{d_{p}^{2}\varepsilon }{32\mu RT\tau \delta }P_{\mathrm{ave}}\Delta P$$

where *P*_ave_ is the average pressure, Pa.

If *Kn* is between 0.05 and 50, both Knudsen diffusion and Poiseuille flow must be considered. In this case, the flow can be calculated as:

(9) $$J=\left(\frac{d_{p}\varepsilon }{3RT\tau \delta }{\left(\frac{8RT}{\pi M}\right)}^{0.5}+\frac{d_{p}^{2}\varepsilon }{32\mu RT\tau \delta }P_{\mathrm{ave}}\right)\Delta P$$

In the VMD process using a hydrophobic membrane for desalination, the governing mass transfer mechanism is generally the combination of Knudsen diffusion and Poiseuille flow (Equation (9)) [21].

Heat transfer in the VMD module includes the conductive heat transfer in the ceramic membrane, the convective and conductive heat transfers of the feed in the tube side, the latent heat transfer for the water vapor. The model equations can be written according to the Boussinesq approximation [16,26]:

(10) $$\rho_{f}C_{f}u\cdot \nabla T=\nabla \cdot \left(k_{f}\nabla T\right)$$

(11) $$\rho_{f}u\cdot \nabla u=-\nabla P+\nabla \cdot \left[\mu \left(\left(\nabla u+\nabla u^{T}\right)-\frac{2}{3}\nabla \cdot uI\right)\right]+\rho_{f}g$$

(12) ∇u=0

where *ρ_f_*, *C_f_*, *k_f_*. and *μ* are the density, heat capacity, and thermal conductivity of the feed flow, respectively; *T* is the local temperature; *u* is the local velocity; *P* represents the local pressure; *r* is the radial direction; *μ* is the viscosity of the feed flow.

Since a high vacuum degree exists in the permeate side of the VMD membrane module, heat conduction through the membrane can be neglected, so heat transfer for VMD can be written as [16]:

(13) $$-k_{f}\frac{\partial T}{\partial r}=J\cdot \nabla H+\frac{k_{m}\cdot \left(T_{fm}-T_{pm}\right)}{\delta }$$

The boundary conditions are as follows:

(14) $$T\left(r,0\right)=T_{0};\;\frac{\partial T}{\partial r}\left(r,0\right)=0;\;u\left(r,0\right)=u_{0};\;\;\frac{\partial u}{\partial r}\left(r,0\right)\;=0$$

where *k_m_* is the thermal conductivity of the membrane; *T_fm_* and *T_pm_* are the temperatures of the membrane surface on the feed side and on the permeate side, respectively.

### 3.2. Nmerical Solution

The proposed 2D model equations were solved by COMSOL Multiphysics which is widely accepted as a very powerful software for solving partial differential-nonlinear algebraic equations based on the finite element method (FEM) [8,27,28,29]. Internally within the COMSOL software, two kinds of physical field were coupled to simulate the membrane distillation process, including the Laminar Flow, the Darcy’s Law, and the Heat Transfer in Solids and Fluids.

### 3.3. Materials

Five specifications of tubular dual-layer ceramic membranes, which have the same macroscopically internal/external diameters of 8/12 mm but different pore sizes and thicknesses of the top and/or support layers, were supplied by Membrane Industrial Park, Jiangsu, China. Hexadecyltrimethoxysilane (≥ 85 vol%, Aladdin Biochemical Technology Co., Ltd., Shanghai, China) and ethanol (≥ 99.7 vol%, Wuxi City Yasheng Chemical Co., Ltd., Wuxi, China) were used for the hydrophobic modification of the ceramic membranes.

### 3.4. Preparation and Characterization of the Hydrophobic Membrane

The modifier was prepared by adding concentrated hexadecyltrimethoxysilane (indicated hereafter as C16) to ethanol to concentration of 0.1 mol/L at room temperature for 24 h. The dried commercial membranes were immersed into the C16 solution at 35 °C for 12 h. During this process, the –OCH_3_ group in the silane molecule undergoes hydrolysis to form silanol (R–Si–(OH)_3_), which possesses hydrophobicity [12,30]. The membranes were taken out and washed with deionized water and then dried at 120 °C for 4 h. The membranes were stored at room temperature.

The water contact angle of these ceramic membranes was measured by a contact angle analyzer (Dataphysics-OCA20, DataPhysics Instruments GmbH Co., Ltd., Filderstadt, Germany). The five modified membranes had stable contact angles of 132°–135°, indicating they exhibited satisfactory hydrophobicity. The pore size distribution of the ceramic membranes was determined by a mercury intrusion pore size analyzer (Poremaster GT-60, Anton Paar, Shanghai, China). The porosity was measured using the Archimedes method at room temperature [31]. The tortuosity can be roughly estimated as the reciprocal of porosity [32]. The characteristics of the five hydrophobic membranes are presented in Table 2.

The structure parameters of the grafted membranes were found to be very close to those of the raw membranes provided by the manufacturer. This was likely due to the fact that the grafted C16 layer on the inner surface of the pore channels was very thin, with a thickness < 2 nm [30,33]. The value was much smaller than the pore sizes (≥ 100 nm for the top layer and ≥ 1.0 μm for the support layer) of the membranes used in this work. Therefore, the hydrophobic modification had an insignificant effect on gas permeation.

### 3.5. Vacuum Membrane Distillation Experiment

**Figure 8:**
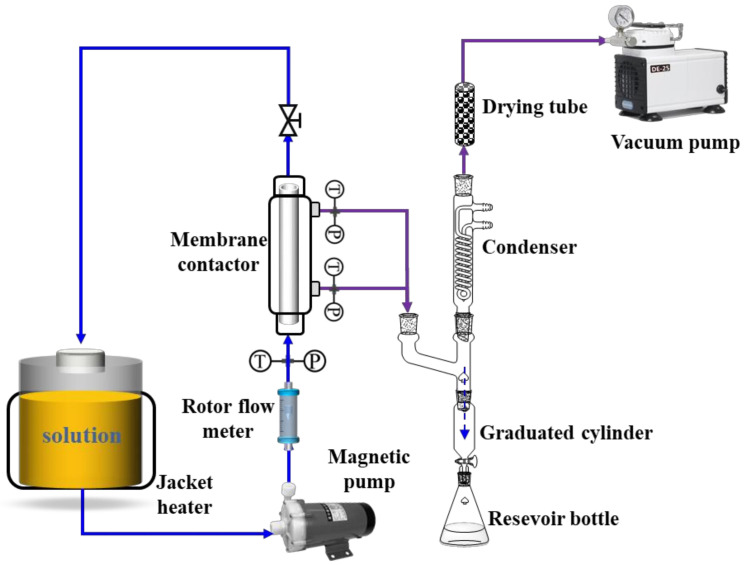
Schematic diagram of the membrane distillation process.

The experimental apparatus presented in Figure 8 was used for vacuum membrane distillation. All experiments were carried out in a 304 stainless membrane contactor with inner diameter of 18 mm and effective length of 110 mm (using a short module to keep the flows isothermal on both sides of the membrane). Water in a heating tank was continuously fed into the lumen side of the membrane contactor at a liquid flow of 160 L/h (under turbulence state, corresponding Reynolds number of ~7500) to minimize the temperature difference between the liquid bulk and the liquid–vapor interface. The liquid flow rate was controlled and measured by a rotameter (accuracy: ± 2%). The feed temperature and pressure conditions were monitored using thermal sensors (0−200 °C, PT100-type, Hangzhou Sinomeasure Automation Technology Co., Ltd., China) and pressure transmitters (0−0.6 MPa, SIN-P300, Hangzhou Sinomeasure Automation Technology Co., Ltd., China), respectively. Additionally, the permeable side pressure of the membrane contactor was generated by a vacuum pump and determined by a pressure transmitter (−0.1–0 MPa, SIN-P300, Hangzhou Sinomeasure Automation Technology Co., Ltd., China). The vapor was extracted from the membrane contactor and then condensed. The condensate volume was determined by a precise graduated cylinder. The experimental permeate flux can be calculated by using Equation (15).

(15) $$J=\frac{\Delta m}{A\cdot \Delta t}$$

where *J* is the permeate flux, kg∙m^−2^∙s^−1^; *m* is the weight of the condensed water, kg; *A* is the inner surface of the ceramic membranes or the liquid–gas interfacial area, m^2^; *t* is time, s.

The parameters of the membrane contactor and the operational conditions are presented in Table 3.

## 4. Conclusions

In summary, we have shown that the proposed 2D model equations enabled a reliable simulation of water permeate flux for the VMD process with tubular asymmetric ceramic membranes. Moreover, this paper also highlights the feasibility of applying the innovative permeation correlation to simplify calculations for vapor transport through asymmetric ceramic membranes by assuming that those membranes, consisting of two or three layers, present, effectively, a quasi-homogeneous layer and by introducing the relative correction factors. Our research findings indicate that correction factors should be useful for the design and optimization of membranes’ microstructure and geometry.

## Figures and Tables

**Table 1:** Specifications of the membrane contactor and operational conditions.

Specifications	Value
Membrane characteristics	
Tube inner diameter, *R_t_in_* (mm)	4 ~ 8
Overall membrane thickness, *δ_overall_* (mm)	1 ~ 2
Thickness of top layer, *δ_top_* (mm)	0.02 ~ 0.5 for two-layer membranes; 0.005 ~ 0.05 for three-layer membranes;
Thickness of transition layer, *δ_tran_* (mm)	0 for two-layer membrane; 0.01 ~ 0.2 for three-layer membrane;
Thickness of support layer, *δ_sup_* (mm)	*δ_overall_*–*δ_top_*–*δ_tran_*–*δ_sup_*
Pore size of top layer, *d_top_* (nm)	50 ~ 500 for two-layer membrane; 5 ~ 20 for three-layer membrane;
Pore size of transition layer, *d_tran_* (nm)	0 for two-layer membrane; 100 for three-layer membrane;
Pore size of support layer, *d_sup_* (nm)	500 ~ 5000
Porosity	0.4
Tortuosity	2.5
Operational conditions	
Temperature, T (K)	333 ~ 373
Permeate side pressure, P (KPa)	10 ~ 50

**Table 2:** Structure parameters of five hydrophobic asymmetric membranes.

Schematic		Codes	The Top Layer				The Support Layer			
			*d*_I_ (nm)	*δ*_I_ (μm)	*ε*_I_	*τ*_I_	*d*_II_ (μm)	*δ*_II_ (mm)	*ε*_II_	*τ*_II_
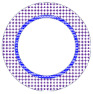		M1	480	20	0.36	2.8	3.2	2.0	0.33	3.0
		M2	150	20	0.36	2.8	3.2	2.0	0.33	3.0
		M3	150	20	0.36	2.8	1.1	2.0	0.38	2.6
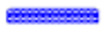	Top layer	M4	150	60	0.36	2.8	1.1	2.0	0.38	2.6
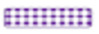	Support layer	M5	100	40	0.36	2.8	1.0	2.0	0.4	2.5

**Table 3:** Membrane contactor parameters and operational variables.

Parameters	Values
Module inner diameter (mm)	18
The outer and inner diameters of the Membranes (mm)	12/8
Effective length of the membrane (mm)	110
Feed temperature (K)	328–348
Water feed flow rate (L/h)	150
Permeate side pressure (kPa)	50

## Data Availability

Not applicable.

## Sample Availability

Samples of the compounds are not available from the authors.
